# Unveiling Complexity: A Case Report of Catastrophic Antiphospholipid Syndrome With Coronary Occlusion and a Concomitant Patent Foramen Ovale Shunt

**DOI:** 10.7759/cureus.48456

**Published:** 2023-11-07

**Authors:** Nava R Sharma, Sudarshan Gautam, Prabal KC, Sajog Kansakar, Marlon E Rivera Boadla, Madalasa Pokhrel, Arjun Basnet, Saral Lamichhane

**Affiliations:** 1 Medicine, Manipal College of Medical Sciences, Pokhara, NPL; 2 Internal Medicine, Maimonides Medical Center, Brooklyn, USA; 3 Internal Medicine, Rasuwa District Hospital, Rasuwa, NPL; 4 Internal Medicine, Manipal College of Medical Sciences, Pokhara, NPL; 5 Internal Medicine, Montefiore Medical Center, New Rochelle, USA; 6 Cardiology, Tower Health Medical Group, West Reading, USA; 7 Internal Medicine, Gandaki Medical College, Pokhara, NPL

**Keywords:** systemic lupus erythema, patent foramen oval, acs, apla syndrome, apla

## Abstract

Antiphospholipid syndrome (APLS) and systemic lupus erythematosus (SLE) are rare autoimmune disorders that afflict a small percentage of the global female population. The complexity of these conditions is further exacerbated by their propensity to give rise to recurrent thrombosis and obstetric morbidity, thereby posing intricate challenges for clinicians and patients alike. One of the most concerning aspects of these diseases is the heightened risk they confer for accelerated atherosclerosis, which can ultimately culminate in the development of acute coronary syndrome (ACS). This case report describes a 27-year-old female with APLS, SLE, and lupus nephritis. She suffered from a catastrophic antiphospholipid syndrome (CAPS) episode and simultaneously developed ACS. She also had a patent foramen ovale (PFO) shunt.

## Introduction

Antiphospholipid syndrome (APLS), a complex autoimmune disorder characterized by recurrent thrombosis and obstetric morbidity, presents a significant challenge for clinicians and patients due to its multifaceted nature [1]. APLS and systemic lupus erythematosus (SLE) are rare autoimmune disorders that affect a small fraction of the global female population, impacting fewer than 5% [2]. Individuals afflicted with these conditions face an elevated risk of an accelerated progression of atherosclerosis, which can potentially result in the development of acute coronary syndrome (ACS) [3].

This case report illuminates an extraordinary clinical scenario in which the convergence of APLS, SLE, and ACS has led to an unprecedented medical challenge. We present the case of a 27-year-old female patient with an exceptional medical history who experienced catastrophic antiphospholipid syndrome (CAPS), accompanied by ACS and a concomitant patent foramen ovale (PFO) shunt, leading to a series of events that tested the limits of modern medical management.

## Case presentation

A 27-year-old female with a past medical history of APLS, SLE, and lupus nephritis was transferred from another hospital following a cardiac arrest. She had been diagnosed with ACS/non-ST elevation myocardial infarction (ACS/NSTEMI) with a 100% occlusion of the right coronary artery three days before the cardiac arrest, for which she had undergone a mechanical thrombectomy without stent placement. Return of spontaneous circulation (ROSC) was achieved after a single round of cardiopulmonary resuscitation. Post-ROSC, she remained unconscious with some response to painful stimuli and exhibited non-purposeful movements. Therapeutic hypothermia management was initiated but later deferred due to her hypercoagulable state.

**Figure 1:**
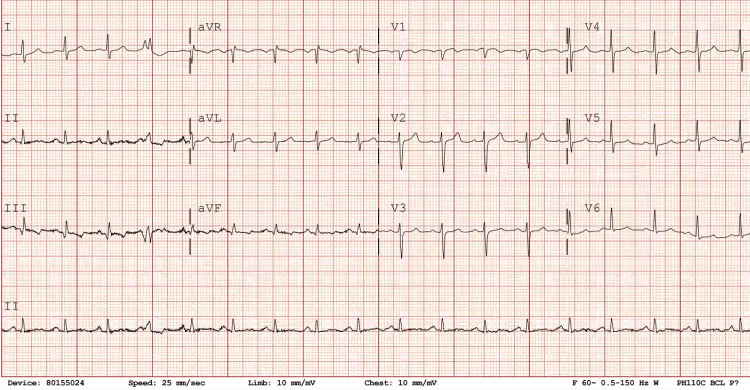
An initial EKG revealed ST depression and Q wave with T-wave inversion in inferior leads.

An initial electrocardiogram (EKG) revealed ST depression and Q wave with T-wave inversion in inferior leads, as shown in Figure 1. A transthoracic echocardiogram (TTE) on the same day revealed a soft tissue mass measuring approximately 1.84 cm x 1.18 cm on the aortic side of the right coronary cusp, likely a thrombus. The TTE also indicated moderate right ventricular enlargement, reduced right ventricular systolic function, an akinetic right ventricular free wall, and a moderately elevated pulmonary artery systolic pressure of 61 mmHg (normal range: approximately 15-30 mmHg). The left ventricular ejection fraction (LVEF) was measured at 55% (normal range: 55-70%). A cranial CT scan showed no remarkable findings. A pulmonary artery catheter was placed, revealing elevated filling pressures and preserved cardiac output.

The patient was initiated on aspirin, clopidogrel, a heparin drip, and milrinone for right ventricular failure. Subsequent measurements from the pulmonary artery catheter indicated a low cardiac index and elevated systemic vascular resistance. The patient was successfully weaned off pressors and inotropes and was extubated. An episode of acute blood loss anemia led to a suspension of dual antiplatelet therapy and anticoagulation. Milrinone was discontinued due to tachycardia with elevated blood pressure post-extubation. Pulmonary artery measurements continued to show high systemic vascular resistance, and in the context of acute kidney injury, the patient was started on a calcium channel blocker (nifedipine). The use of hydralazine was deferred due to concerns about potential drug-induced lupus.

**Figure 2:**
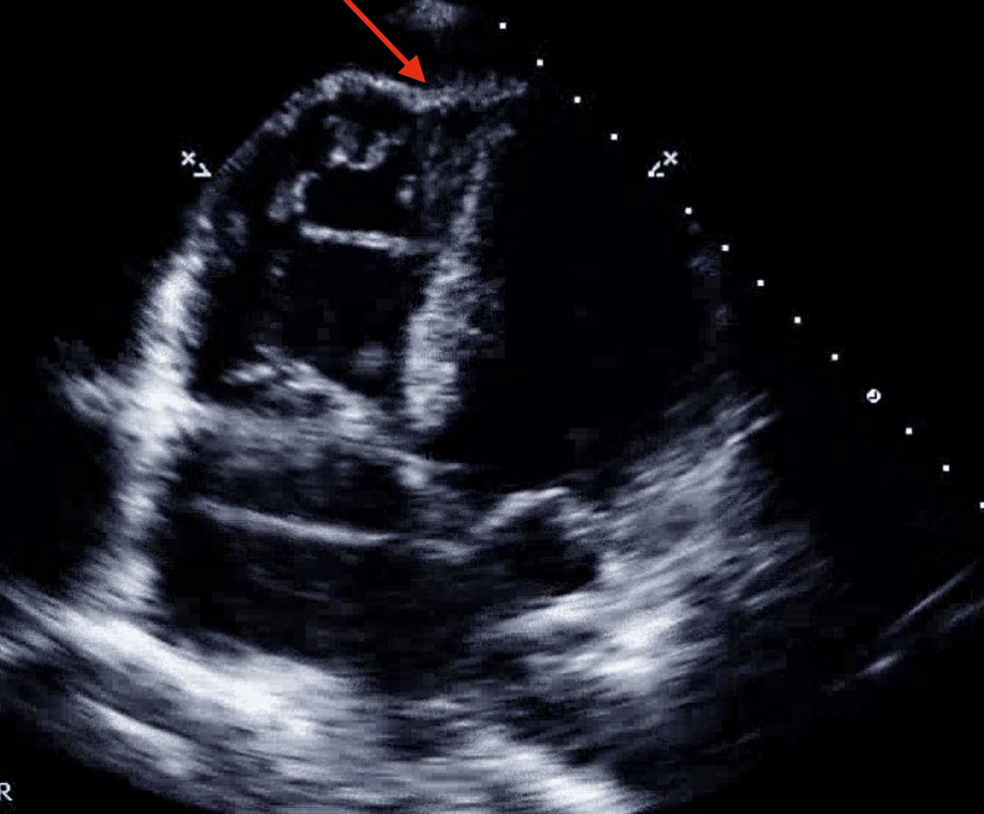
A bedside TTE revealed a possible McConnell sign, as shown by the red arrow. TTE: transthoracic echocardiogram

**Figure 3:**
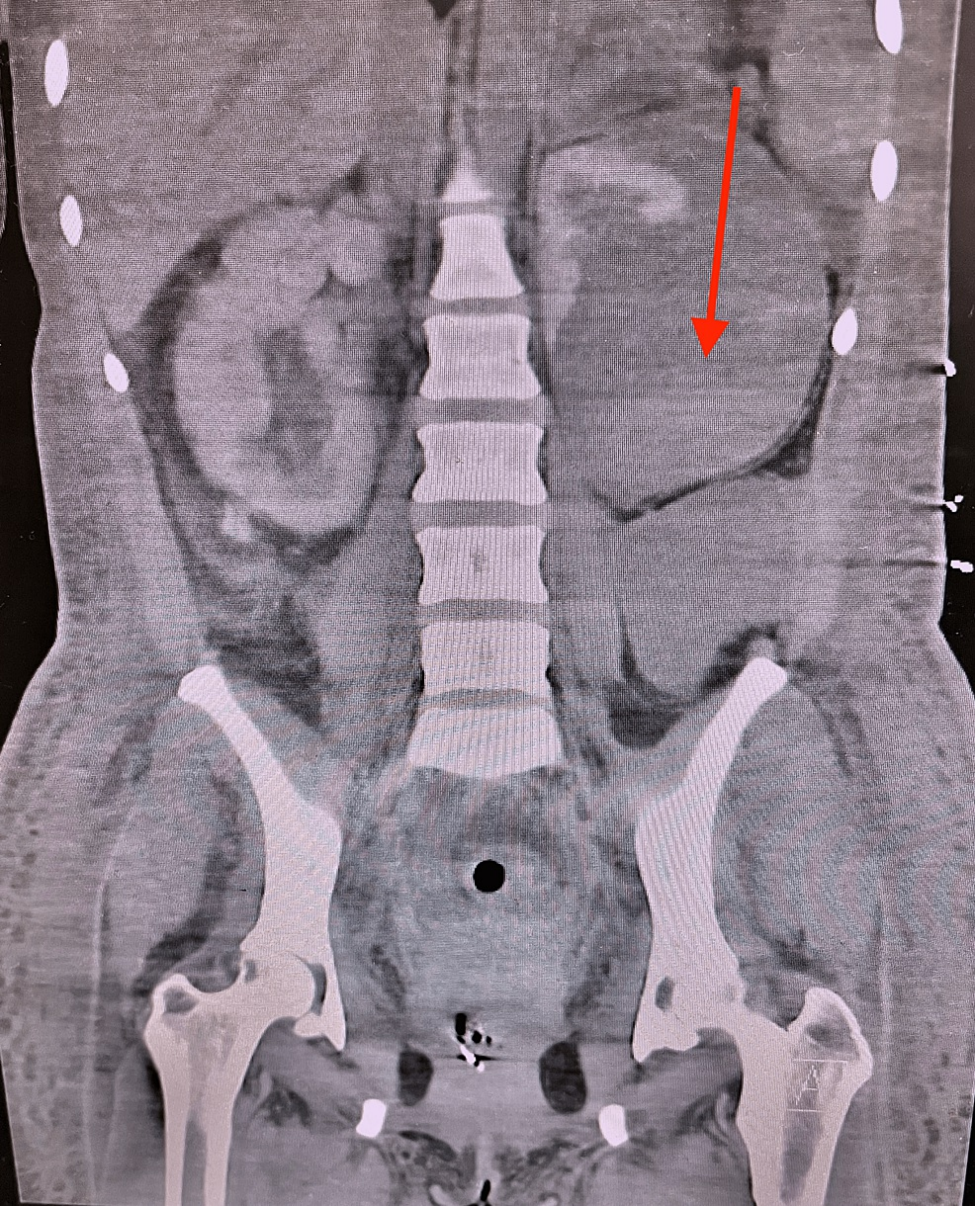
Computed tomography angiography of the chest, abdomen, and pelvis showed a stable-sized renal hematoma, as shown by the red arrow.

On the fourth day of admission, the patient exhibited tachycardia, hypertension, tachypnea, and hypoxia. A bedside TTE revealed a possible McConnell sign (the paradoxical movement can be seen as a "w-shape" or "d-shape" motion of the right ventricle free wall during systole, where the middle portion does not contract effectively, but the basal and apical portions of the right ventricle do contract), as shown in Figure 2. At the same time, a computed tomography angiography of the chest, abdomen, and pelvis showed a stable-sized renal hematoma, as shown in Figure 3. As a result, anticoagulation was resumed due to suspected pulmonary embolism (PE), and the patient was started on a nitroglycerin drip. Further anemia necessitated a blood transfusion. A drop in hemoglobin levels was attributed to microangiopathic hemolytic anemia, as indicated by the presence of schistocytes on the peripheral blood smear, elevated lactate dehydrogenase, and positive anticardiolipin, double-stranded DNA, and ribonucleoprotein antibodies. This pattern suggested CAPS, leading to consultations with rheumatology and hematology specialists. Pulse-dose steroids and intravenous immunoglobulin were initiated, given a high suspicion of lupus vasculitis.

**Figure 4:**
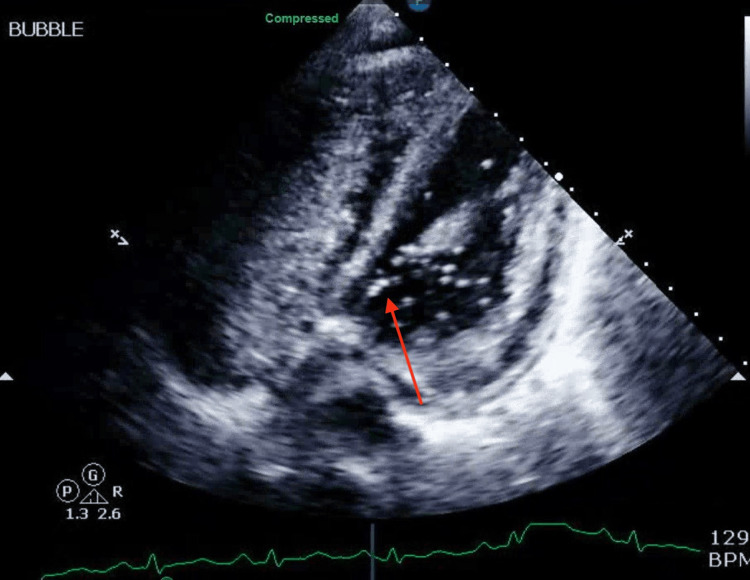
An echo with a bubble study indicated a right-to-left shunt, which suggested a patent foramen ovale (PFO), as shown by the red arrow.

Due to deteriorating respiratory status and hemodynamic instability, the patient was re-intubated and started on vasopressors. Oxygen saturation declined, necessitating a titration of FiO2 to 100%. Despite receiving maximum oxygen supplementation, the patient remained persistently hypoxic. A bedside echo with a bubble study indicated a right-to-left shunt, which suggested a PFO, as shown in Figure 4. Nitric oxide and sildenafil were introduced to address elevated pulmonary artery pressure, and milrinone was restarted for ionotropic support, which improved her hypoxia. Discussions with interventional cardiology occurred regarding mechanical circulatory support devices, but these interventions were deemed unsuitable due to multiple thrombotic events and hypercoagulability. Plasmapheresis was postponed due to hemodynamic instability.

Hematology and rheumatology consultations did not find indications for rituximab or cyclophosphamide, mainly due to concerns about possible infection, normal antibodies, and the patient's critical condition. A repeat TTE showed an improved LVEF, a reduced right-to-left shunt, and a smaller echogenic mass on the right coronary cusp. Milrinone and nitric oxide were tapered and discontinued.

A right heart catheterization (RHC) performed while the patient was on sildenafil showed mildly elevated right-sided filling pressures, mild pulmonary hypertension (PH), and good cardiac output. A normal pulmonary angiogram ruled out chronic thromboembolic PH. The patient remained hemodynamically stable, and steroids were reduced per the recommendations of the rheumatology team. Furosemide was initiated for right-sided heart failure. Warfarin was bridged with argatroban and continued for APLS. The patient developed left leg dry gangrene, likely due to catastrophic APLS, but the family declined leg amputation.

Upon discharge, the patient was prescribed long-term warfarin with instructions to monitor the international normalized ratio (INR) to assess the anticoagulation effect. The patient was scheduled for follow-up with rheumatology, hematology, and cardiology for further management.

## Discussion

APLS can be categorized into primary APLS and secondary APLS. The latter is often linked to connective tissue diseases. SLE is the leading cause of secondary APS among the various connective tissue diseases. Research suggests that in patients with SLE, the prevalence of antiphospholipid antibodies (APLAs), such as lupus anticoagulant (LA) or anticardiolipin antibodies (ACLAs), can range as high as 30% to 50% [4].

CAPS, characterized by the rapid onset of multiple organ failure due to widespread thrombosis, is a severe form of APLS and presents a clinical problem for healthcare providers [5]. One of the critical challenges in this case was the coexistence of APLS and SLE, as these autoimmune disorders often overlap and potentiate each other's clinical manifestations. The development of ACS in a young patient with CAPS is rare, emphasizing the need for increased awareness of potential cardiovascular complications in this population [6].

Another significant challenge was managing multiple thrombotic events, including right coronary artery occlusion, pulmonary embolism, and microangiopathic hemolytic anemia. These events occurred in the context of the patient's hypercoagulable state, which complicated the selection and titration of anticoagulation and antiplatelet therapies. The presence of a patent foramen ovale shunt further complicated the clinical picture by allowing right-to-left shunting of emboli and contributing to hypoxia. Management decisions, such as initiating nitric oxide, sildenafil, and milrinone, aimed to address the patient's deteriorating oxygenation. The management team also faced challenges in determining the appropriate use of invasive interventions and immunosuppressive therapies. Mechanical circulatory support devices were considered but ultimately deemed unsuitable due to the heightened risk of thrombosis. The decision to defer plasmapheresis due to the patient's hemodynamic instability exemplifies the intricate balance of risks and benefits in such cases.

In the management of CAPS, anticoagulation therapy plays a pivotal role. The initiation and titration of anticoagulation regimens are paramount but must be approached with caution, considering the patient's hypercoagulable state and potential bleeding risks [7]. Recent guidelines have emphasized the importance of tailoring anticoagulation therapy to the specific presentation of CAPS, considering the extent and type of thrombotic events and the presence of comorbidities [6]. As seen in this case, therapeutic decisions require clinical judgment and adaptability, such as deferring therapeutic hypothermia due to hypercoagulability and adjusting anticoagulant dosing following an acute blood loss anemia episode.

The role of immunosuppressive therapies in CAPS management remains a subject of debate [8]. In this case, consultations with hematologists and rheumatologists did not yield clear indications for rituximab or cyclophosphamide, reflecting the complex nature of CAPS. These agents should be considered cautiously, especially in critically ill patients with potential contraindications. Recent studies and expert consensus recommend a risk-stratified approach, with immunosuppressive therapy reserved for those with the highest refractory CAPS risk [7,9]. At the same time, other patients may benefit from anticoagulation alone [7,9].

The presence of a PFO, in this case, introduced an additional layer of complexity. PFOs are recognized, but their clinical significance can vary. In some instances, as observed here, PFOs can serve as conduits for emboli. It is worth considering that the patient's RCA occlusion and the development of dry gangrene in the leg may not necessarily be due to paradoxical emboli; they could also potentially result from issues, such as left ventricular thrombus. A PFO prompted a multidisciplinary discussion involving cardiologists and critical care specialists to consider nitric oxide, sildenafil, and milrinone for managing pulmonary hypertension and right-to-left shunting [10]. The treatment of PFO-related complications emphasizes the need for a tailored approach based on individual patient characteristics and clinical presentation.

Finally, the challenge of CAPS in young patients with overlapping autoimmune disorders highlights the need for further research and evidence-based guidelines to improve patient outcomes. Such cases' rarity and multifaceted nature make it difficult to establish standardized treatment protocols. Ongoing research into the pathophysiology and management of CAPS is essential to provide clinicians with the tools to address these cases' intricacies successfully.

## Conclusions

The case report underscores the necessity for further investigation and the development of evidence-based guidelines to steer the treatment of intricate scenarios involving APLS, lupus, and ACS. It also underscores the significance of promptly recognizing and appropriately addressing complications, such as pulmonary emboli, right-sided heart failure, and severe pneumonia, to enhance patient outcomes. This case report illustrates the intricate nature of CAPS when coexisting with ACS and a PFO shunt. Effective management demands a multidisciplinary approach, a comprehensive understanding of the underlying pathophysiology, and the capacity to tailor therapeutic approaches to each patient's unique needs. While challenges persist, ongoing research and clinical expertise continue to illuminate the optimal strategies for managing these complex cases.
